# Shock Wave Lithotripsy in Ureteral Stones: Evaluation of Patient and Stone Related Predictive Factors

**DOI:** 10.1590/S1677-5538.IBJU.2014.0330

**Published:** 2015

**Authors:** Ozgur Yazici, Murat Tuncer, Cahit Sahin, Mehmet K. Demirkol, Alper Kafkasli, Kemal Sarica

**Affiliations:** 1 Dr. Lutfi Kirdar Kartal Training and Research Hospital, Istanbul, Turkey

**Keywords:** Ureteral Calculi, Lithotripsy, Body Mass Index

## Abstract

**Purpose:**

To evaluate the patient and stone related factors which may influence the final outcome of SWL in the management of ureteral stones.

**Materials and Methods:**

Between October 2011 and October 2013, a total of 204 adult patients undergoing SWL for single ureteral stone sizing 5 to 15 mm were included into the study program. The impact of both patient (age, sex, BMI,) and stone related factors (laterality, location, longest diameter and density as CT HU) along with BUN and lastly SSD (skin to stone distance) on fragmentation were analysed by univariate and multivariate analyses. Results: Stone free rates for proximal and distal ureteral stones were 68.8% and 72.7%, respectively with no statistically significant difference between two groups (p=0.7). According to univariate and multivariate analyses, while higher BMI (mean: 26.8 and 28.1, p=0.048) and stone density values (mean: 702 HU and 930 HU, p<0.0001) were detected as statistically significant independent predictors of treatment failure for proximal ureteral stones, the only statistically significant predicting parameter for the success rates of SWL in distal ureteral stones was the higher SSD value (median: 114 and 90, p=0.012).

**Conclusions:**

Our findings have clearly shown that while higher BMI and increased stone attenuation values detected by NCCT were significant factors influencing the final outcome of SWL treatment in proximal ureteral stones; opposite to the literature, high SSD was the only independent predictor of success for the SWL treatment of distal ureteral stones.

## INTRODUCTION

Following its clinical introduction by Chaussy et al. (1) in 1980 extracorporeal shock wave lithotripsy (SWL) became the most common treatment modality with its safe and successful results in renal as well as ureteral stones (2). However, failure of SWL may cause unnecessary exposure of the treated kidney and neighbouring organs to high energy shock waves which may result in tissue damage. Identification and the use of these predictive factors in clinical setting will both increase the efficacy and decrease the cost by reducing the number of unnecessary treatment sessions as well as hospital visits.

Regarding the parameters evaluated so far, many studies did clearly demonstrate that success rates may be related to both patient (body mass index=BMI, skin to stone distance =SSD), and stone related factors (location, longest diameter and density in CT hounsfield unit =HU) (3–8). However, majority of these studies have mostly examined the outcomes of kidney stones treated with SWL. To our knowledge, these factors have not been evaluated enough for the success rate of ureterolithotripsy with SWL in the literature.

In this present study, we aimed to analyse the possible predictive factors detected by pre-procedural unenhanced abdominopelvic computed tomography (NCCT) to assess the success rates of SWL in the management of ureteral stones.

## MATERIALS AND METHODS

Between October 2011 and 2013, a total of 204 adult patients undergoing SWL for ureteral stone with a longest diameter of 5 to15mm were evaluated in a prospective manner. All patients had a single non-impacted radiopaque ureteral stone evaluated with kidneys, ureter, and bladder (KUB) radiography and NCCT. Exclusion criteria were multiple ureteral stones, anatomically solitary kidney, patients who could not tolerate SWL due to pain, congenital abnormality, pre-SWL JJ stent in place, renal insufficiency, previous ureteral surgery, previous SWL of a stone in the same ureter. 204 patients fulfilling the criteria were included into the study programme. Prior to the treatment in all cases stone location (proximal: from ureteropelvic junction to distal sacroiliac joint; and distal: distal to sacroiliac joint) and size was evaluated by NCCT. Complete urine test, if needed urine culture and antibiogram test, simple biochemical and coagulation tests were performed in all patients before SWL. The impact of patient related factors (age, sex, BMI) and stone related factors (laterality, location, longest diameter and HU), blood urea nitrogen (BUN) and lastly SSD on fragmentation were analysed.

**Figure 1:**
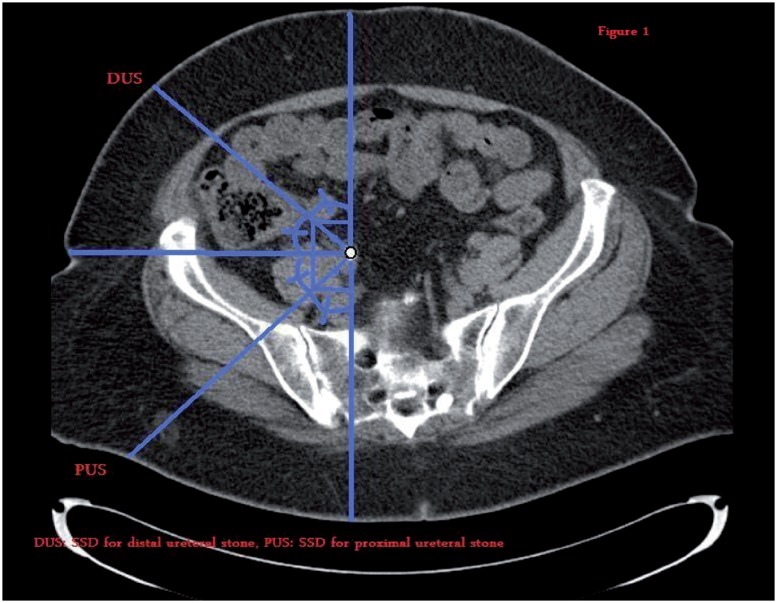
Showing pathways for ultrasonic waves.

The BMI was calculated by dividing the weight (kg) by square of the height (m). NCCT with 5mm contiguous sections at 120kw and 90mA was performed in all patients with a multidetector row helical CT scanner (Somatom Plus; Siemens, Germany). The longitudinal stone dimension was calculated by multiplying the collimation thickness and the number of images in which the stone could be seen. The transverse dimension was chosen as the diameter of stone from image showing its largest width. Maximum dimension of the stone was accepted as either the longitudinal or the transverse diameter, whichever had the highest value measured. The average NCCT attenuation value as the representative HU was measured by drawing a region of interest smaller than the stone in the image showing the stone in the largest dimension (Figure-1). The SSD was calculated by measuring the distance from skin to the stone at posterolateral and anterolateral 45° for proximal and distal ureteral stones (Figure-1), respectively.

SWL was performed with an electromagnetic lithotripter Compact Sigma (Dornier Med Tech System GmbH, Wessling, Germany) by same operator. It was performed in supine position and with a standardized treatment angle. All sessions of SWL were performed under IM diclofenac Na or methimazole Na (if Cr was high) injection medication. All stones were disintegrated under fluoroscopic guidance. On the other hand, there was a difference in technique between SWL treatment of proximal and distal ureteral stones where shock waves were relegated to stone from posterior and anterior aspect for proximal and distal ureteral stones, respectively. In our opinion, that was why pelvic bone did not cause problems during SWL session. Each session has been completed either after application of a total of 3000 shock waves or until the stone was completely disintegrated. The degree of shock wave power (PW) delivered during SWL was recorded as 1 to 6 and the shock wave frequency was 90/minute. Individual power setting was adjusted according to patients’ tolerance. Patients were evaluated 1 week after each session with KUB film and repeat treatment was performed if there was a stone fragment. The maximal session number for a patient to say that the stone was resistant to SWL was 3. No patients had medical expulsive therapy (MET) before or after SWL sessions. In the light of radiographic assessment with NCCT after 3 months following the last lithotripsy session, patients were categorized either as the stone free (SF) group if there was no detectable residual stone fragment and the residual stone (RS) group if there was.

Both univariate (chi-square or t-test) and multivariate (logistic regression or analysis of covariance=ANCOVA) tests were performed to determine statistically significant independent factors. One way analysis of variance (ANOVA) test was performed to analyse if there were or not HU and SSD differences between patients requiring different number of SWL session to be SF. If parameters did not show normal dispersion, Mann-Whitney U test which is the nonparametric equal of t test and Kruskal Wallis test which is the nonparametric equal of one way ANOVA test were performed. Pearson or Spearman correlation tests were used to determine the correlation between SSD, the degree of PW delivered and being SF. Statistical analyses were performed using SPSS software v19.

## RESULTS

Of the 204 patients evaluated (153 men and 51 women), 99 had a stone on the right and 105 on the left side. While 160 patients had proximal ureteral stones, 44 had distal ureteral stone with a mean stone size of 8.97± 2.44mm (range: 5 −15mm) in the whole group. Overall mean patient age was 43.0±14.3 year with a mean BMI value of 27.0±3.8 kg/m^2^. The overall mean stone density was 745±303 HU, and the overall mean SSD was 123±25mm. Treatment was unsuccessful in 62 patients (30.4%) all of which underwent semi-rigid or flexible ureterorenoscopy.

Success rates of proximal and distal ureteral stones were 68.8% (n: 110) and 72.7% (n: 32), respectively (p=0.7). However, when the stone characteristics were well examined, it was clear that proximal and distal ureteral stones had different stone features (Table-1). As shown in Table-1, distal ureteral stones had more favourable features for SWL treatment than proximal ureteral stones.

**Table 1:** Proximal and distal ureteral stones' characteristics.

	Proximal	Distal	P
BMI (mean±SD)	27.2±3.9	25.5±2.4	0.008^a^
HU (mean±SD)	773±303	544 ±218	0.001^a^
SSD (mean±SD)	125 ±24	106 ±23	0.001^a^
Stone largest diameter (median and range)	9 (5-15)	7 (5-11)	0.006^b^

a Independent sample t test;

b Mann Whitney U test

According to univariate analyses, while there were two independent predictive factors of SWL outcomes in proximal ureteral stones which were BMI and HU, the only significant predictor for the success of treatment of distal ureteral stones with SWL was SSD (Table-2). HU/mm value was calculated for distal ureteral stones by dividing the HU value to stone largest dimension (mm) because of the fact that small stones have artificially low NCCT stone density. However, we again did not find significant difference between RS (mean: 89±25) and SF (mean: 81± 31) groups for distal ureteral stones (p=0,596). Mean HU values of proximal ureteral stones treated successfully with SWL requiring 1, 2 and 3 sessions of SWL were 633±246, 744±206, 821±295, respectively and there was a statistically significant difference between patients requiring 1 and 3 sessions (one way ANOVA: p for between groups=0,008 and p for 1 and 3=0,011). However, there was no significant difference between SSD values in distal ureteral stones requiring different number of SWL sessions for a successful fragmentation (Kruskal Wallis: p=0.513).

**Table 2:** SWL outcomes for proximal and distal ureteral stones.

	SF group	RS group	P
BMI^p^ (mean±SD)	26.8±3.8	28.1±3.8	0.048^a^
HU^p^ (mean±SD)	702±254	930±343	0.0001^a^
SSD^p^ (mean±SD)	125±23	126±26	0.754^a^
Stone largest diameter^p^ (median and range)	9 (5-15)	10 (5-15)	0.349^b^
BMI^d^ (median and range)	24.8 (21-30)	25.5 (23.6-29.0)	0.767^b^
HU^d^ (median and range)	415 (282-855)	708 (452-778)	0.237^b^
SSD^d^ (median and range)	114 (90-145)	90 (56-110)	0.012^b^
Stone largest diameter^d^ (median and range)	7 (5-11)	8 (6-8)	0.910^b^
SSD^d^ (mean±SD)	113±18	85±24	0.01^a^
SSD^d^ (mean±SD) (effect of PW eliminated)	108±8	99±15	0.285^c^

a Independent sample t test;

b Mann Whitney U test;

c ANCOVA test;

p for proximal ureteral stones;

d for distal ureteral stones

According to multivariate analysis, obesity and HU>765 were found to be the independent predictors of failure for proximal ureteral stones (Table-3). Analysis of the relationship between SSD, SF rates and PW (1 to 6) delivered, did show that there was a direct proportional relationship between SSD, PW and SF rates for distal ureteral stones. When we eliminated the effect of PW on disintegration, we observed that there was no significant difference between SSDs for SF and RS groups (Table-2).

**Table 3:** Multivariate analysis showing relationship between HU, BMI and being SF for proximal ureteral stones.

	B	SE	95% CI	P
BMI ≤ 30 > 30	-1.953	0.429	0.172-0.926	0.032
HU ≤ 765 > 765	-0.918	0.397	0.065-0.309	0.0001

**B =** regression coefficient; **SE =** standard error; **CI =** confidence interval. Logistic regression analysis

## DISCUSSION

Following its clinical introduction in 1980 by Chaussy et al., SWL became the treatment of choice in the majority of urinary stones with safe and effective results both in adults and children (9). Additionally, as a practical and cost effective treatment modality SWL is being applied for more than 90% of the stones in adult patients (10–12) without hospitalization and loss of manpower. The ultimate aim of this treatment modality is the efficient fragmentation and complete clearance of the disintegrated stone fragments. Accumulated experience so far has clearly indicated that the fragmentation and clearance of stone fragments depend on some certain patient (BMI, SSD) and stone related factors (location, longest diameter and HU) (3–8).

BMI and SSD are two important parameters that have been evaluated in details. Although they may have some impact on the efficacy of shock waves, particularly varying distribution of body fat between different genders and race (13) makes it hard to be used as a reliable marker in the prediction of SWL success. Variable data in the literature have been reported regarding the importance of BMI and SSD in predicting the outcome of SWL. While Pareek et al. found BMI to be a significant predictor of success (3); in another trial BMI failed to predict the outcome of SWL, whereas SSD remained to be a significant predictor (14). Additionally one should keep in mind that surrounding tissue around kidney and ureteral stones is different especially for caliceal stones which are primarily encircled by renal parenchyma. In our study, BMI and SSD values were found to be significant predictors of SWL success in the treatment of proximal and distal ureteral stones respectively (Tables 1 and 3). The variable efficacy of high energy shock waves in patients with higher BMI values may be mainly due to the fact that SSD values may not increase in accordance to BMI values in all cases. In other words, BMI and SSD may affect the final outcome of SWL in an independent manner from each other as shown in our cases due to the fact that fatty tissue distribution could be variable from case to case.

Many studies have confirmed that SSD is a significant predictor of SWL outcome for ureteral stones. In a multivariate analysis study, Wiesen-thal et al. did show that SSD (110mm -OR, 0.49) was a significant predictor for lithotripsy success in ureteral stones (15). Perks et al. also further supported this finding by reporting that SSD of <9cm (OR: 2.8) can estimate SWL success (16). In our study, SSD has been found to be a significant predictor for treatment outcome in distal ureteral stones (median for SF group: 114 (90-145) and RS group: 90 (56-110), p=0.012). As demonstrated above, we surprisingly found that median SSD value of SF group was higher than that of recorded in RS group. Further correlation analysis did demonstrate that there was a positive correlation between SSD and SF rate, SSD and PW, PW and SF rate. ANOVA test was used to eliminate the possible effect of PW on the relation between SSD and SF rate. As a result, it was clear that when the effect of PW was eliminated, no statistically significant difference with respect to the effect of SSD could be shown between SF and RS groups (Table-2). This is opposite to the literature data reported for kidney and proximal ureteral stones (17, 18). The answer for the contradictive finding where SSD has been found to be lower in cases with RS than SF cases is that there was a significant positive correlation between SSD and PW applied. This could probably be due to the use of higher PW in cases with higher SSD values where these cases could tolerate pain better than the cases with lower SSD values for distal ureteral stones. However, it could probably be acceptable for a certain upper cut-off level of SSD that has not been assessed yet. It is of course valid for the SWL performed without analgesia as in our hospital and most hospitals in Turkey.

In the light of these findings, we may say that the correlation of SF rate with BMI and SSD may not be as clear and simple as reported in the literature. Because if a case is not treated under analgesia, high SSD may have good effect on SWL treatment for distal ureteral stones and to clarify this issue further we certainly need studies performed with larger series of patients.

Related with the possible effect of HU on the success rates of SWL in ureteral stones in their original study, Joseph et al. (19) observed stones of patients with HU values of <500, 500-1000, >1000 had SF rates of 100%, 86% and 55% along with median shock wave numbers of 2500, 3390 and 7300 respectively. In another study, it was found that the mean HU values were significantly higher in cases with RS group (3, 5). Gupta et al. (20) found that failure was mostly observed in patients with a HU values of >750 where the largest stone diameter has been detected as >11mm. Also, 77% of all these patients required >3 sessions with an overall SF rate of 60%. Similarly, Wang et al. (21) found that SWL was unsuccessful in patients with a HU value of >900 and stone volume of >700mm^3^. Similar to these data, in our study, SF cases had a lower mean HU value than the ones with RS cases treated for proximal ureteral stones (Table-1). However, there was no statistically significant difference with respect to HU values between RS and SF groups in distal ureteral stones. This may be explained well by the limited number of distal ureteral stone patients treated in our group. Logistic regression analysis revealed that both HU and BMI values were independent predictors of SWL outcome for proximal ureteral stones (Table-2).

One of the negative aspects of this study was the use of low-resolution beam collimation (5mm) which caused artificially low NCCT stone density for small stones (22). However, when we compared the HU/mm value calculated by dividing the HU value to stone largest dimension (mm) for RS (mean: 89±25) and SF (mean: 81±31) groups, we again did not find significant difference between them (p=0,596) for distal ureteral stones. Another negative aspect of this study was that we did not fill visual analogue scale (VAS) form for pain of patients submitted to SWL. The other negative aspect of this study was that we could not perform SWL for non-opaque stones due to technical difficulties. Also, low number of distal ureteral stone patient was the other important limitation. Besides, in this study we aimed to investigate the effect of only patient and stone related predictive factors. Because of this reason we did not use MET during SWL sessions which may produce bias on our evaluation of patient and stone related predictive factors. Finally, stone dimension was not found as an independent predictive factor for the success opposite to the literature and this may be the reason of narrow range of stone dimension which is the positivity of our study giving opportunity of analysing other parameters without the effect of it.

## CONCLUSIONS

Our findings have clearly shown that while higher BMI and increased stone attenuation values detected by NCCT were significant factors that influence the final outcome of treatment in proximal ureteral stones with SWL, SSD was the only independent predictor of failure or success for the treatment of distal ureteral stones treated with SWL.

We (all authors) declare that we have no conflict of interest. No company or organization sponsored our study and we don't have any financial relationship with any company or organization.

## ABBREVIATIONS

SWL =: Extracorporeal shock wave lithotripsy
BMI =: Body mass index
SSD =: Skin to stone distance
HU =: CT hounsfield unit
NCCT =: Non-contrast abdominopelvic computed tomography
KUB =: Kidney, ureter, and bladder
BUN =: Blood urea nitrogen
PW =: Power of shock wave
MET =: Medical expulsive therapy
SF =: Stone free
RS =: Residual stone
ANCOVA =: Analysis of covariance
One way ANOVA =: One way analysis of variance
VAS =: Visual analogue scale
DUS =: For distal ureteral stones
PUS =: For proximal ureteral stones
